# Neurofeedback Treatment Affects Affective Symptoms, But Not Perceived Cognitive Impairment in Cancer Patients: Results of an Explorative Randomized Controlled Trial

**DOI:** 10.1177/15347354221149950

**Published:** 2023-01-24

**Authors:** Madeleine Fink, Saskia Pasche, Kira Schmidt, Mitra Tewes, Martin Schuler, Bernhard W. Mülley, Dirk Schadendorf, Norbert Scherbaum, Axel Kowalski, Eva-Maria Skoda, Martin Teufel

**Affiliations:** 1 University of Duisburg-Essen, Essen, Germany; 2 Partner Site University Hospital Essen, and German Cancer Research Center (DKFZ), Essen, Germany; 3 University of Wuppertal, Wuppertal, Germany; 4 University Hospital Essen, Essen, Germany; 5 NeuroFit GmbH, Krefeld, Germany; 6 IB University of Applied Health and Social Sciences, Berlin, Germany

**Keywords:** cancer, neurofeedback, EEG biofeedback, mindfulness, distress, depression, anxiety, self-efficacy, quality of life, alpha band

## Abstract

**Background:**

EEG biofeedback (NF) is an established therapy to enable individuals to influence their own cognitive-emotional state by addressing changes in brainwaves. Psycho-oncological approaches of NF in cancer patients are rare and effects are hardly studied.

**Objective:**

The aim of this explorative, randomized controlled trial was to test the effectiveness of an alpha and theta NF training protocol, compared to mindfulness based therapy as an established psycho-oncological treatment.

**Methods:**

Of initially 62 screened patients, 56 were included (inclusion criteria were cancer independent of tumor stage, age >18 years, German speaking; exclusion criteria suicidal ideation, brain tumor). Randomization and stratification (tumor stage) was conducted by a computer system. Participants got 10 sessions over 5 weeks, in (a) an NF intervention (n = 21; 13 female, 8 male; MAge = 52.95(10 519); range = 31 to 73 years)) or (b) a mindfulness group therapy as control condition (CG; n = 21; ie, 15 female, 6 male; MAge = 50.33(8708); range = 32 to 67 years)). Outcome parameters included self-reported cognitive impairment (PCI) as primary outcome, and secondary outcomes of emotional distress (DT, PHQ-8, GAD-7), fatigue (MFI-20), rumination (RSQ), quality of life (QoL, EORTC-30 QoL), self-efficacy (GSE), and changes in EEG alpha, and theta-beta band performance in the NF condition.

**Results:**

No changes in cognitive impairment were found (*P* = .079), neither in NF nor CG. High affective distress was evident, with 70.7% showing elevated distress and 34.1% showing severe depressive symptoms. Affective symptoms of distress (*P* ≤ .01), depression (*P* ≤ .05) and generalized anxiety (*P* ≤ .05) decreased significantly over time. No differences between NF and CG were found. There was a significant increase of the alpha band (*P* ≤ .05; N = 15) over the NF sessions. Self-efficacy predicted QoL increase in NF with *P* ≤ .001 and an explained variance of 48.2%.

**Conclusion:**

This is the first study to investigate NF technique with regard to basic mechanisms of effectiveness in a sample of cancer patients, compared to an established psycho-oncological intervention in this field. Though there were no changes in cognitive impairment, present data show that NF improves affective symptoms comparably to mindfulness-based therapy and even more pronounced in QoL and self-efficacy.

Trial registration: ID: DRKS00015773

## Introduction

The number of people currently living with cancer in Western Europe has almost doubled in recent decades, in part due to a decline in cancer mortality.^1-4^ The decline in cancer mortality has contributed to increased life expectancy. The number of cancer survivors and long-term cancer patients has increased even more than the number of new cases in highly developed regions. In Europe alone, there were an estimated 3.45 million new cancer cases and 1.75 million cancer deaths in 2012.^1^ As a result, there are likely about 32 million people worldwide who have had cancer in their lifetime.^3^ According to deMoor et al^2^ there were about 13.7 million cancer survivors living in the United States. Sixty-four percent of this population survived 5 years or longer, 40% survived 10 years or longer, and 15% survived 20 years or longer after diagnosis. Cancer diagnosis and treatment place an enormous burden on many affected individuals and their families. A cancer diagnosis usually evokes negative emotional states such as denial, anxiety, depression, and anger.^5^ Other than that, cancer and its treatment, for example, chemotherapy, can lead to numerous clinical symptoms, that might also last during remission.^6^ Many cancer patients experience fatigue, cognitive impairment, pain, anxiety, and depression. A drug-free and non-invasive intervention for these psychological and physiological symptoms is neurofeedback (NF) therapy, which is still rarely used in psycho-oncology approaches. By changing electroencephalographic measured amplitudes of one’s own brain activity and cognitive processes, NF has the potential to alleviate multiple long-term symptoms of cancer survivors and improve their quality of life (QoL).^6^ Despite several limitations, Alvarez et al^7^ demonstrated that NF is an effective intervention for cancer patients and leads to improvement in fatigue and cognitive symptoms. A previously published review from our research group was able to show the effectiveness of NF interventions in adults on cancer-specific symptoms, such as pain, fatigue, cognition, depression, and sleep disturbances.^8^

An RCT pilot study of 20 female breast cancer patients showed that women who received a 4-week alpha NF intervention had both improved short-term memory and increased QoL.^9^ A waitlist study of our working group by Schmidt et al^10^ found a positive effect of a 5-week alpha and theta NF intervention on health-related QoL and self-efficacy in cancer patients. Furthermore, the results indicate a lower QoL but higher profit of the intervention in younger patients compared to over-55-year-old cancer patients.

Neurophysiological target parameters such as arousal, emotional valence, and sleep are associated with certain spectral frequency bands in the EEG. An increase in arousal has been associated with an increase in central frontal beta band,^11^ whereas a decrease is related to a higher central frontal theta band.^12^ Especially depressive symptoms are related to certain target parameters of NF,^13^ which thus become relevant for psycho-oncological patients. Stewart et al^14^ found an association between an increased alpha band in the left compared to the right frontal cortex and an increased susceptibility to negative emotions, which seems to be a risk marker for major depression. Over parietal and temporal areas, a psycho-oncological case study on visual neuropathic symptoms suggested that the observed alpha increase after NF training might be associated with the improvement of cancer-related symptoms.^15^ Results on pain reduction could be found in a headache patient case study,^16^ fibromyalgia,^17^ trigeminal neuralgia,^18^ and complex regional pain syndrome type I.^19^

Other than these promising results, our working group showed that research focusing on NF in cancer patients is lacking, and unfortunately, many publications about the efficacy/effectiveness of NF in mental and brain disorders show severe methodological weaknesses, enhancing the need to close this research gap.^8^ Moreover, no comparisons were found with other drug-free interventions, like mindfulness-based stress reduction programs which are established and often used in the treatment of cancer. According to this systematic review article, it is of great interest to compare NF with other approaches like mindfulness-based therapy.^8^

Therefore, the present study aims to compare the effects of an alpha/theta NF training protocol with a mindfulness therapy, which is already highly used in the treatment of cancer patients.^20^ Similar to NF, mindfulness therapy shows an increased alpha band and theta power compared to a resting state with closed eyes.^20,21^ Like NF, mindfulness has positive effects on cancer-related symptoms, such as depression and pain.^22^ A reduction of affective symptoms, such as rumination in dysphoric mood, due to mindfulness trainings has already been found in cancer patients/patients of different entities.^23,24^ Although mindfulness-based therapy is widely used, low acceptance and isolated side effects have been reported.^25^

Based on these findings, we expect an effect of NF and mindfulness on specific clinical symptoms in a sample of cancer patients by changing alpha and theta-beta activity. If the effectiveness of NF is comparable to mindfulness, it might be implemented in the clinical context as an additional psycho-oncological intervention for in- and outpatient cancer patients.

## Method

### Study Design and Participants

The study was designed as a randomized, controlled, clinical study with a single-blinded waitlist and parallel group paradigm. The RCT was registered at the German Clinical Trials Registry (ID: DRKS00015773) and approved by the Ethics Committee of the Medical Faculty of the University of Duisburg-Essen (No.: 18-8079-BO dated 18.10.2018). Recruitment took place in the *West German Cancer Centre Essen*. Inclusion criteria were age between 18 and 70 years and the diagnosis of a malignant tumor disease. Exclusion criteria were a major depressive episode (F32.2 or F33.2, F33.3) according to the ICD-10 checklist,^26^ acute suicidality, psychotic symptoms or illness, central nervous disorders, and poor language competence. After participants had given their written informed consent, the anamnestic interview was conducted. The assessment of various questionnaires took place before a 5 weeks waitlist (in accordance with other investigations in this field, eg, Alvarez et al^7^) (*t*0), before each intervention (*t*1), and after the 5-week intervention (*t*2). At time point *t*1 participants were randomized (and stratified by a central computer system (Microsoft Excel randomization functioning) according to tumor stage (UICC)) into either the experimental group, which received a NF intervention, or the control group (CG). The CG received a mindfulness group intervention that followed a manual consisting of evidence-based mindfulness exercises. Up to this point, the patients were blinded. To exclude experimenter effects, the main examiner conducted both interventions.

Completers were defined a priori as patients who completed 6 or more of the 10 intervention sessions. None of the patients was non-completers. However, one patient dropped out of the study after the sixth NF session due to somatic deterioration. This patient’s data could not be included in the analyses.

### Neurofeedback Intervention

**Figure 1.**
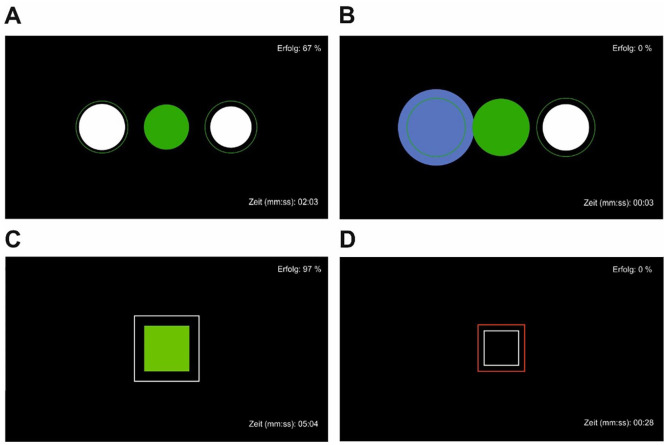
Neurofeedback stimuli as displayed for the patients. (A) Achieving target alertness. (B) Target alertness not achieved. (C) Achieving target attention. (D) Target attention not achieved.

Mobile NF training was performed using a modified *Mind Wave* headset (NeuroSky Inc., 2011), and the electrode was positioned at coordinate Cz. The measured EEG signals were sent via WLAN from the headset to the computer and recorded and processed in BioEra Pro (Proatech LLC) and Processing (Processing Foundation) for visualization. Filtering algorithms based on the *Fast Fourier Transform* were used to decompose the raw signals into individual frequency bands. This output signal was used as feedback, which was displayed on the monitor as the target (“Erfolg” engl. success) condition during training. As stimuli, patients saw geometric figures that changed depending on the degree of correspondence with the target condition (Figure 1) on a monitor with a distance of 1.5 m. The stimuli were either circles or squares that changed color from green and white to blue and red indicating high and low compliance with the target condition, respectively. The success rate was displayed in the upper right corner and the elapsed time during the exercise in the lower right corner of the screen.

The experimenter (MF) was present throughout the training but did not provide verbal feedback or make any manipulations to the feedback process. The NF intervention included a minimum of 6 and a maximum of 10 training sessions, each lasting 35 to 40 minutes. The length and duration of the NF was based on the few already existing findings^8^ on NF interventions in cancer patients ranging from 10 to 100 sessions twice a week. In this study each session had the following structure:

- *Resting state for about 5 minutes*,
- *Alpha-bandwidth training (9*-*13 Hz) and theta / beta (*>*20 Hz) reduction for 10 minutes*,
- *Resting state for about 5 minutes*,
- *Target: Theta/Beta-Ratio ≤2,5 for about 5 minutes*,
- *Resting state for about 5 minutes, and*
- *Second alpha-bandwidth training (9*-*13 Hz) and theta/beta (*>*20 Hz) reduction for 10 minutes.*

#### Processing of the frequency-based data of the NF training

The data sets of the NF training sessions were each filtered using 0.3 to 40 Hz bandpass and artifacts were removed and prepared for further processing and Fourier power analysis. The corresponding values of the frequencies were given in 0.5 Hz steps and each from 1 to 40 Hz; the spectral average over 10 seconds was obtained in each case. For subjects 42 and 52 there were no entries in time intervals. Therefore, the corresponding data were not evaluated. (Detailed evaluations were not available for the following subjects and data sets (sessions): 004_01, 004_02, 004_03, 004_07, 007_03, 008_01, 008_02, 008_05, 011_05, 013_06, 016_02, 016_06, 016_09, 021_06, 024_06, 025_03, 025_06, 025_07, 025_08, 029_04, 029_06, 030_06, 037_01, 037_09, 038_02, 038_034, 038_06, 040_01, 040_02, 047_05, 047_06, 047_07, 052_01).

### Mindfulness Intervention

Similar to the NF intervention, the manual based, mindfulness-based group training took place twice a week for a period of 5 weeks. The therapy sessions lasted 35 to 40 minutes with groups of 2 to 6 patients. The manual was based on evidence-based mindfulness programs and consisted of psychoeducation and 10 versatile mindfulness exercises.^27-29^

A psychoeducational part focused on the possible psychological consequences of a cancer diagnosis, including the subsequent emotions, coping strategies and psychosomatic processes. Various physical, social, and environmental stressors were discussed that could lead to individual physiological and psychological responses that could mix with cancer symptoms and result in a downward cycle and low quality of life. An approach through a non-judgmental, patient and accepting attitude toward oneself was discussed. In the first session, basic information about mindfulness was provided as well as a 10-minute sitting meditation as a basic exercise. Thereafter, each session began with the basic exercise, followed by another exercise aimed at developing skills such as letting go of ruminating thoughts, accepting negative emotions, and staying in an observing mindfulness role. Some coordination and breathing exercises focused on a physiological impulse and were processed in the group through facial miming expressions and balancing exercises.

### Assessment Instruments

#### Primary outcome

##### Functional assessment of cancer therapy—Cognitive function [FACT-Cog].^30^

The Fact-Cog was used as primary outcome based on the previous investigation by Alvarez et al^7^ The Fact-Cog is a validated questionnaire to assess perceived cognitive deterioration and related QoL in cancer patients. It contains 4 scales: perceived cognitive impairments (20 items), impact on quality of life (4 items), comments from others (4 items) and perceived cognitive abilities (9 items). The items are scored from 0 (“never”) to 4 (“several times a day”), with higher scores indicating a better health status. All item scores of the scales perceived cognitive impairments, impact on quality of life and comments from others have been reversed as proposed by the authors.^30^

#### Secondary outcomes

##### Multidimensional fatigue-inventory [MFI-20].^31^

The 20-item MFI-20 is designed to measure fatigue and shows good internal consistency and construct validity. It has already been tested in a large German sample of cancer patients, confirming the factorial structure of the MFI-20.^32^ All items are answered using a five-point Likert scale ranging from 1 (“yes, that is true”) to 5 (“no, that is not true”). It provides 5 scores for different dimensions of fatigue: general fatigue, physical fatigue, reduced activity, reduced motivation, and mental fatigue. Higher scores are associated with more acute levels of fatigue.

##### Distress thermometer.^33^

The widely used and highly validated Distress Thermometer allows the patient to assess their distress level of the past week on a visual analog scale from 0 (“no distress”) to 10 (“the worst distress imaginable”). Scores of 5 or higher indicate a high distress level.

##### Patient health questionnaire depression scale [PHQ-8].^34^

The PHQ-8 is one scale of the PHQ, containing 8 items that are answered on a 4-point Likert scale from 0 (“not at all”) to 3 (“nearly every day”). It was designed to assess the severity of depressive disorders. For the PHQ-8, it is assumed that values between 10 and 14 indicate a moderate severity, 15 to 19 a severe severity and 20 to 27 highest severity.^34^

##### Generalized anxiety disorder scale-7 [GAD-7].^35^

The 7-item GAD-7 is another valid and efficient scale of the PHQ, which screens for generalized anxiety disorder and assesses its severity on a 4-point Likert scale from 0 (“not at all”) to 3 (“nearly every day”). When assessing generalized anxiety using GAD-7, scores of ≥5, ≥10, and ≥15 are considered mild, moderate, and severe generalized anxiety, respectively.^35^

##### Ruminative response scale of the response styles questionnaire [RSQ].^36^

The RSQ was designed to assess the response styles rumination and distraction, both are possible reactions to dysphoric emotion. The 21-item rumination scale includes self-focus, symptoms, their possible consequences and causes. All items are assessed on a 4-point Likert scale ranging from 1 (“almost never”) to 4 (“almost always”), with higher scores indicating a more frequent use of the response style.

##### European organization for research and treatment of cancer core quality of life questionnaire [EORTC QLQ-C30].^37^

The 30-item EORTC QLQ-C30 was designed to measure the self-rated QoL in cancer patients. It is widely used in clinical studies in the oncological research field. It provides a global QoL score, 5 functional scales, with higher scores indicating a better level of functioning, as well as 9 symptom scales, for which higher scores correspond to a higher level of symptoms. Two items rate the global QoL score and are answered on a 7-point Likert scale from 1 (“very poor”) to 7 (“excellent”). The rest of the items are scored from 1 (“not at all”) to 4 (“very much”).

##### General self-efficacy scale [GSE].^38^

The GSE is based on the concept of perceived self-efficacy introduced by Bandura as part of his social-cognitive theory. The 10 items measure the general positive expectations concerning overcoming difficult situations, while attributing the success to one’s own competences. All items are scored from 1 (“not at all true”) to 4 (“exactly true”). A higher score corresponds to a greater individual’s generalized sense of self-efficacy.

### Statistical Analysis

Statistical analysis was performed using the Statistical Program for Social Sciences SPSS version 26 (IBM, New York). Figures were created using CorelDRAW X5 (Corel, Ottawa) and Prism 9.0.2 (GraphPad, San Diego).

For all analyses, the significance level was set at α = .05. Before performing calculations, data were corrected for statistical outliers using graphical analysis (boxplots) for ±1 standard deviation (SD) for psychometric data; in the case of differences of variables (Δ*t*1 − *t*0 or Δ*t*2 − *t*1) and frequency-based data in the experimental intervention (NF) for ±2 standard deviations. Descriptive statistics are shown in Supplemental Table 1. Here, group comparisons were made based on the presence of non-normal distribution using Wilcoxon-signed-rank tests and Mann-Whitney-*U* tests. ANOVAS with repeated measures (rmANOVAS) were calculated for the frequency-based data in the experimental intervention subgroup over the mean 60 seconds of the respective training for the first 6 training sessions (time points). Prevalences of the affective measures were determined for the total sample. In addition, rmANOVAS with intervention (NF vs CG), age, and gender as covariates were conducted. Because the study was conducted as a clinical RCT, it could not be controlled for tumor type. Tumor types were so heterogeneous that no influence of a single type could be expected. Tumor stages were stratified at randomization. In the case of non-normally distributed data, non-parametric procedures were used or, if sphericity was not present, Greenhouse-Geisser correction was used. Post hoc tests were performed using Bonferroni correction. Group comparisons between therapy interventions Δ*t*2 − *t*1 and waitlist (WL-CG, Δ*t*1 − *t*0) were performed using Kruskal-Wallis-Tests and Monte-Carlo correction. Post-hoc age effects were calculated by median split (55> × <55 years) using Wilcoxon-signed-rank tests. SD is shown below in parentheses () after mean. Error bars show the 95% confidence interval.

## Results

### Participants

**Figure 2.**
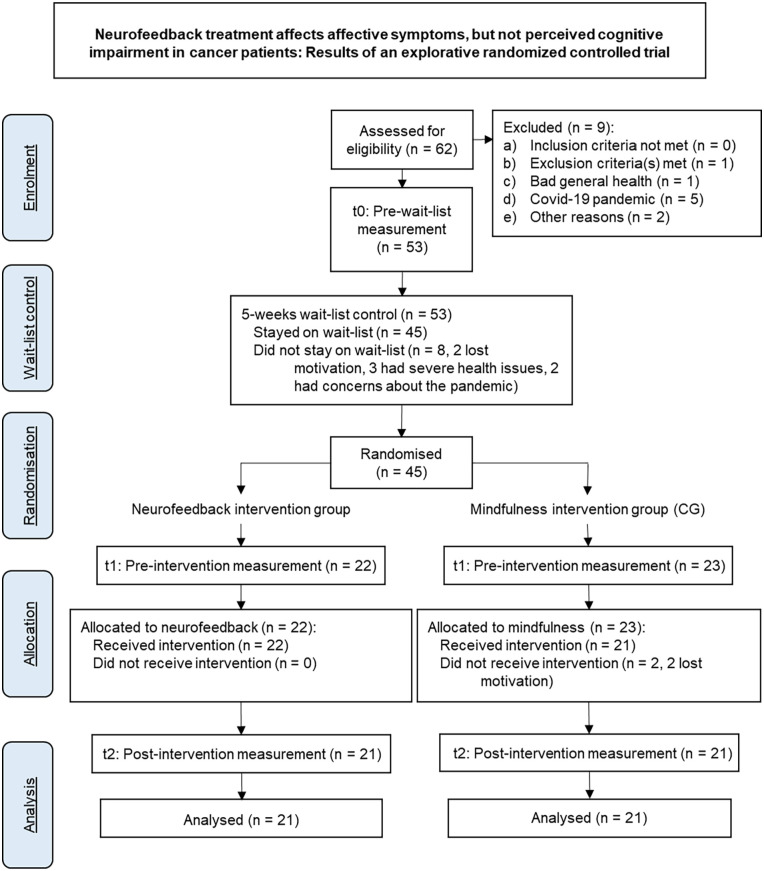
CONSORT flowchart.

Recruitment took place in the *West German Cancer Centre Essen* from October 25, 2018 to April 15, 2021. Out of 62 initially interested patients, 56 were included and one patient was excluded due to an existing alcohol dependence syndrome (F10.2).^26^ The drop-out rate was 25% partly due to the COVID-19 pandemic, so that 28 female patients (*M* = 50.07 years; SD = 9.014; range = 31-61 years) and 14 male patients (*M* = 54 years; SD = 10.379; range = 32-73 years) underwent the RCT. One patient reported an incorrect age at the time of inclusion; data from this 73-year-old patient were not excluded from the analyses in accordance with the intention-to-treat principle. The corresponding CONSORT flowchart^39^ is shown in Figure 2. The demographic data for the total cohort as well as for the 2 randomized, stratified subcohorts (NF and CG) can be found in Table 1.

**Table 1.** Demographics.

	Total N = 42 (%)			
	Waitlist	Neurofeedback	Mindfulness	Statistics
	N = 42 (%)	N = 21 (%)	N = 21 (%)	
Sex				
Female	28 (66.7) 76.5	13 (61.9)	15 (71.4)	*U*(41) = −0.647; *P* = .518
Male	14 (33.3) 23.5	8 (38.1)	6 (28.6)	
Age				
Mean (SD; range) [years] (SD. range)	51.64 (9.629; 31-73)	52.95 (10.519; 31-73)	50.33 (8.708; 32-67)	*U*(41) = 176.0; *P* = .262
Median	53.5	55	51	
Relationship				
Alone	10 (23.8) 23.	4 (19)	6 (28.6)	
With partner	32 (76.2) 76.5	17 (81)	15 (71.4)	
Employment status				
Working	17 (40) 58.	10 (47.6)	7 (33.3)	
On sick leave	16 (38)	5 (23.8)	11 (52.4)	
Retired/incapacitated	4 (9) 17.6	3 (14.3)	1 (4.8)	
Unemployed	1 (2) 17.6	1 (4.8)	2 (9.5)	
Other	4 (9) 5.9	2 (9.5)	7 (33.3)	
Education				
High school diploma	24 (55) 53.0	11 (52.5)	13 (61.9)	
Secondary school degree (“*Realschule*”)	10 (23)	4 (19)	6 (28.6)	
Secondary school degree (“*Hauptschule*”)	5 (11) 11.8	3 (14.3)	2 (9.5)	
Missing	3 (7.1) 11.8	3 (14.3)		
Cancer type				
Breast	10 (23.8)	6 (28.6)	4 (19)	
Melanoma	8 (19.0)	4 (19)	4(19)	
Lung	4 (9.5)	3 (14.3)	1 (4.8)	
Lymphoma	4 (9.5)	1 (4.8)	3 (14.4)	
Pancreas	3 (7.1)	2 (9.5)	1 (4.8)	
Multiple myeloma	3 (7.1)	2 (9.5)	1 (4.8)	
Head-neck-tumor	2 (4.8)	1 (4.8)	1 (4.8)	
Leukemia	2 (4.8)		2 (9.5)	
Angiosarcoma	1 (2.4)	1 (4.8)		
Rectum	1 (2.4)		1 (4.8)	
Intestine	1 (2.4)		1 (4.8)	
Gall-bladder	1 (2.4)		1 (4.8)	
Seminoma	1 (2.4)	1 (4.8)		
Ovarian	1 (2.4)			
Tumor stage (UICC)				
I	4 (9.5)	3 (14.3) 1 (4.8)	1 (4.8)	
II	3 (7.1)	1 (4.8)	2 (9.5)	
III	18 (42.9)	8 (38.1)	10 (47.6)	
IV	17 (40.5)	9 (42.9)	8 (38.1)	
Median	3	3	3	
Mean (SD)	3.14 (0.916)	3.10 (1.044)	3.19 (0.814)	*U*(41) = 220.5; *P* = 1.0

### Frequency Bandwidth Changes by the NF Therapy

**Figure 3.**
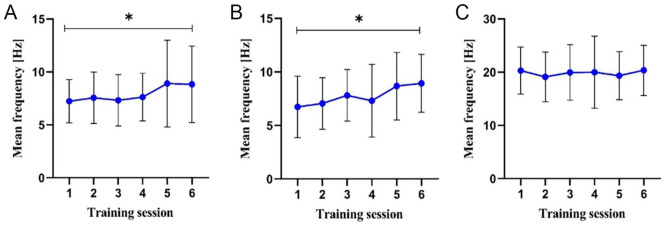
Mean values of the target parameter during the first 6 NF sessions over the mean 60 seconds of the (A) first and (B) second alpha-training (8-13 Hz), and (C) theta/beta-training (Ratio 2.5).

Figure 3 (and Supplemental Table 2) shows the mean values of the target parameters [Hz] during the first 6 NF training sessions (completers) over the mean 60 seconds of the respective training.

The rmANOVA shows a significant time effect for the first alpha band training over the 6 measurement time points, *F*(2.288, 1.81) = 3.356, *P* = .043, Eta² = 0.205. This is also evident for the second training in the paradigm, *F*(2.686, 0.095) = 2.995, *P* = .048, Eta² = 0.176. Post-hoc tests show a significant difference between first and sixth sessions, respectively (*P* = .031 and *P* = .011).

The rmANOVA shows no effect for theta-beta training, <20 Hz (ratio of 2.5), across the 6 measurement time points, *F*(2.437, 0.096) = .869, *P* = .448, Eta² = 0.063.

### Changes in Cognitive Impairment, Fatigue, and Affective Symptoms Due to the NF Therapy in Comparison to the CG

#### Primary outcome: Perceived cognitive impairment

Data on subjectively perceived cognitive impairments (*PCI*) measured by the subscale of the *FACT-Cog*^30^ are first compared with a normative psycho-oncology sample: Normative data for a similar European cohort show a mean PCI score of 56.8 (11.2; range = 26-72).^40^ The present cohort shows significantly lower levels of perceived cognitive impairment compared with the comparison cohort in simple *t*-tests across all 3 measurement time points (see Supplemental Table 3).

**Figure 4.**
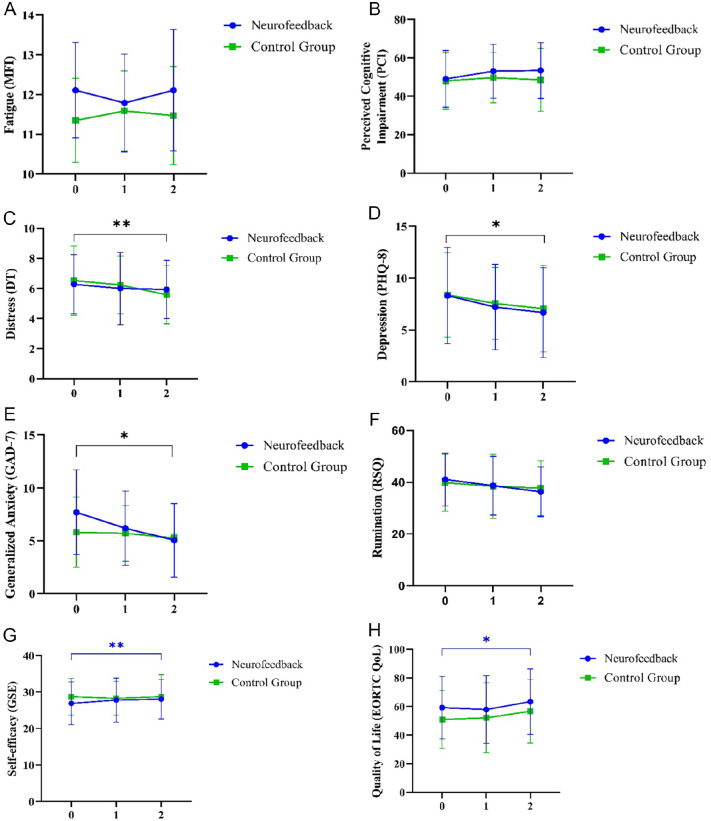
ANOVAS with repeated measures (rmANOVAS) within intervention (Neurofeedback (NF) vs mindfulness control group (CG)) over *t*0, *t*1, and *t*2. (A) The rmANOVA showed no significance for subjectively perceived cognitive impairment across the 3 measurement time points. (B) The rmANOVA showed no significance for mental fatigue. (C) The rmANOVA showed a significant time effect for the psychological distress parameter (distress thermometer) across the 3 measurement time points, *F*(2, 29) = 6.098, *P* = .006, Eta² = 0.296. No intervention effect was found. Pairwise comparisons showed a significant difference between measurement time points *t*0 and *t*3 (Δ*M* = −0.717, *P* = .05). (D) The rmANOVA showed a significant time effect for the depression (PHQ-8) parameter across the 3 measurement time points, *F*(2, 33) = 5.124, *P* = .012, Eta² = 0.237. There was no intervention effect. Pairwise comparisons showed a significant difference between measurement time *t*0 and *t*3 (Δ*M* = −1.478, *P* = .014). (E) The rmANOVA showed no significance for rumination (RSQ). (F) The rmANOVA showed a significant time effect for generalized anxiety, *F*(2, 33) = 4.888, *P* = .014, Eta² = 0.229. A trend for the intervention could be determined showing a greater decrease in GAD values for the NF intervention, *F*(2, 33) = 2.548, *P* = .094, Eta² = 0.134. Pairwise comparisons showed a significant difference between measurement time *t*0 and *t*3 (Δ*M* = −1.603, *P* = .006). (G) The rmANOVA for the experimental group showed a significant increase of QoL due to the NF, *F*(2, 18) = 3.593, *P* = .038, Eta² = 0.166. No significance in the CG. (H) The rmANOVA for changes in self-efficacy in the NF showed significance, *F*(2, 18) = 5.730, *P* = .007, Eta² = 0.241. No significance in the CG.

rmANOVA shows no main effect for subjectively perceived cognitive impairment across the 3 measurement time points, *F*(2, 35) = 2.625, *P* = .079, Eta² = 0.068 (*M*(*t*0) = 48.51 (14.574), *M*(*t*1) = 51.41 (13.573), *M*(*t*2) = 51.03 (15.418), N = 39), Figure 4A). Also, there is no interaction effect for the intervention, *F*(2, 35) = 0.841, *P* = .435, Eta² = 0.023 (*M*_NF_(*t*0) = 49.05 (14.759), *M*_NF_(*t*1) = 53.05 (14.051), *M*_NF_(*t*2) = 53.4 (14.478), *N*_NF_ = 20, *M*_CG_(*t*0) = 47.95 (14.759), *M*_CG_(*t*1) = 49.68 (13.129), *M*_CG_(*t*2) = 48.53 (16.362), *N*_CG_ = 19)). There are no differences between NF and CG, as well as to the waitlist (*H* = 0.202, *P* = .9, see Table 2).

**Table 2.** Group Differences Between Neurofeedback, Mindfulness Control Group, and Waitlist Control.

	Kruskal-Wallis-H	df	*P*	Confidence interval	
				Lower CI	Upper CI
PCI	0.202	2	.900	0.892	0.908
MFI	1.256	2	.544	0.531	0.557
DT	0.611	2	.749	0.738	0.760
PHQ-8	0.392	2	.819	0.809	0.829
RSQ	1.167	2	.562	0.549	0.574
GAD-7	2.576	2	.278	0.266	0.289
EORTC	5.279	2	.073	0.066	0.080
GSE	0.296	2	.866	0.858	0.875

Abbreviations: PCI, Perceived Cognitive Impairments measured by FACT-Cog; MFI, Mental Fatigue measured by MFI-20; DT, Distress Thermometer; PHQ-8, Depression measured by Patient Health Questionnaire Depression Scale; RSQ, Rumination measured by Ruminative Response Scale of Response Styles Questionnaire; GAD-7, Generalized Anxiety measured by Generalized Anxiety Disorder Scale-7; EORTC, Cancer-related Life Quality measured by European Organization for Research and Treatment of Cancer Core Quality of Life Questionnaire (EORTC QLQ-C30); GSE, Self-efficacy assessed with General Self-Efficacy Scale.

#### Fatigue

The rmANOVA shows no significance for mental fatigue, *F*(2, 32) = 0.689, *P* = .509 (*M*(*t*0) = 11.75 (1.18), *M*(*t*1) = 11.69 (1.117), *M*(*t*2) = 11.81 (1.411), N = 36), Figure 4B). There is no intervention effect, *F*(2, 31) = 0.388, *P* = .681, Eta² = 0.024, *M*_NF_(*t*0) = 12.11 (1.197), *M*_NF_(*t*1) = 11.79 (1.228), *M*_NF_(*t*2) = 12.11 (1.524), *N*_NF_ = 19, *M*_CG_(*t*0) = 11.35 (1.057), *M*_CG_(*t*1) = 11.59 (1.004), *M*_CG_(*t*2) = 11.47 (1.231), *N*_CG_ = 17. There is no age effect, as well as no gender effect, over time. There are no group differences (*H* = 1.256, *P* = .544, see Table 2).

#### Distress

The majority of patients (68%-74%) suffered from high levels of distress across the measurement time points (see Supplemental Table 4).

The rmANOVA shows a significant time effect for the psychological distress parameter (distress thermometer) across the 3 measurement time points, *F*(2, 29) = 6.098, *P* = .006, Eta² = 0.296 (*M*(*t*0) = 6.4 (2.103), *M*(*t*1) = 6.11 (2.153), *M*(*t*2) = 5.77 (2.34), N = 35, Figure 4C). No intervention effect is found (*M*_NF_(*t*0) = 6.19 (2.04), *M*_NF_(*t*1) = 6.00 (2.401), *M*_NF_(*t*2) = 5.89 (2.644), *N*_NF_ = 18, *M*_CG_(*t*0) = 6.3 (2.342), *M*_CG_(*t*1) = 6.37 (2.033), *M*_CG_(*t*2) = 5.53 (2.27), *N*_CG_ = 15). There is a significant age effect over time, *F*(2, 29) = 4.885, *P* = .015, Eta² = 0.252. Pairwise comparisons show a significant difference between measurement time points *t*0 and *t*3 (*ΔM = −*0.717, *P* = .05). No group differences appear (*H* = 0.611, *P* = .749, see Table 2). Descriptively, it appears that younger people (<55, median split) start with a higher DT (*M* = 6.68 (1.985) vs *M* = 5.75 (2.306)) and older people show a smaller decrease of DT in the intervention.

#### Depression

Approximately one-third of the cohort (34.1% (*t*0), 26.3% (*t*1), and 29.3% (*t*2)) report a score ≥10 for depression (see Supplemental Table 5).

The rmANOVA shows a significant time effect for the depression parameter across the 3 measurement time points, *F*(2, 33) = 5.124, *P* = .012, Eta² = 0.237 (*M*(*t*0) = 8.66 (4.773), *M*(*t*1) = 7.55 (3.867), *M*(*t*2) = 6.9 (4.636), N = 35, Figure 4D). There is no intervention effect (*M*_NF_(*t*0) = 8.2 (4.549), *M*_NF_(*t*1) = 7.55 (4.186), *M*_NF_(*t*2) = 6.6 (4.235), *N*_NF_ = 19, *M*_CG_(*t*0) = 8.39 (4.075), *M*_CG_(*t*1) = 7.56 (3.468), *M*_CG_(*t*2) = 7.06 (4.165), *N*_CG_ = 18). There is a significant age effect, *F*(2, 33) = 5.124, *P* = .029, Eta² = 0.194). Pairwise comparisons show a significant difference between measurement time points *t*0 and *t*3 (*ΔM = −*1.478, *P* = .014).

The rmANOVA for the NF, in contrast to the CG, shows significance for repeated measures of depression, NF: *F*(2, 17) = 4.764, *P* = .015, Eta² = 0.219 (*M*_NF_(*t*0) = 8.32 (4.643), *M*_NF_(*t*1) = 7.21 (4.117); *M*_NF_(*t*2) = 6.68 (4.334), *N*_NF_ = 19), CG: *F*(2, 15) = 1.213, *P* = .325, Eta² = 0.139 (*M*_CG_(*t*0) = 8.39 (4.075), *M*_CG_(*t*1) = 7.56 (3.468), *M*_CG_(*t*2) = 7.06 (4.165), *N*_CG_ = 18).

No differences appear between the groups (*H* = 0.392, *P* = .819, see Table 2).

Age effect analyses show that younger patients start the NF intervention with a higher PHQ-8 score (*M*(*t*1) = 7.33 (2.841), *M*(*t*2) = 5.67 (4)) and show the greatest remission (significantly different from the older ones, *Z* = −2.035, *P* = .042; *d* = 2.366, *M*_NF_ < 55 = −1.667 (2.291), *N*_NF_ < 55 = 9, *M*_NF_ > 55 = 0.5 (2.261), *N*_NF_ > 55 = 10)). No significance for CG is found.

#### Rumination

The rmANOVA shows no significance for rumination (RSQ), *F*(2, 35) = 0.658, *P* = .509, Eta² = 0.036 (*M*(*t*0) = 40.46 (10.458), *M*(*t*1) = 38.62 (11.729), *M*(*t*2) = 37.10 (9.999), N = 39), Figure 4E). There is no intervention effect, *F*(2, 35) = 0.579, *P* = .031, Eta² = 0.024 (*M*_NF_(*t*0) = 41.11 (10.181), *M*_NF_(*t*1) = 38.74 (11.323), *M*_NF_(*t*2) = 36.37 (9.604), *N*_NF_ = 19, *M*_CG_(*t*0) = 39.85 (10.941), *M*_CG_(*t*1) = 38.5 (12.395), *M*_CG_(*t*2) = 37.80 (10.561), *N*_CG_ = 20). Moreover, there is no age effect as well as no gender effect over time. Also, no group differences are found (*H* = 1.167, *P* = .562, see Table 2).

#### Generalized anxiety

Before inclusion in the study 26.2% of the present cohort report elevated scores of generalized anxiety, after the waiting list period 10.5% do (see Supplemental Table 6).

The rmANOVA shows a significant time effect for generalized anxiety across the 3 measurement time points, *F*(2, 33) = 4.888, *P* = .014, Eta² = 0.229 (*M*(*t*0) = 7.55 (4.424), *M*(*t*1) = 6.12 (3.503), *M*(*t*2) = 4.92 (3.702), N = 35, Figure 4F). A trend for the intervention can be determined showing a greater decrease in GAD value for the NF therapy, *F*(2, 33) = 2.548, *P* = .094, Eta² = 0.134 (*M*_NF_(*t*0) = 7.72 (3.923), *M*_NF_(*t*1) = 6.62 (3.930), *M*_NF_(*t*2) = 5.05 (3.486), *N*_NF_ = 20, *M*_CG_(*t*0) = 5.82 (3.340), *M*_CG_(*t*1) = 5.71 (2.640), *M*_CG_(*t*2) = 5.24 (3.993), *N*_CG_ = 17) . There is also a significant age effect, *F*(2, 33) = 3.590, *P* = .039, Eta² = 0.179. Pairwise comparisons show a significant difference between measurement time *t*0 and *t*3 (*ΔM = −*1.603, *P* = .006).

Here, the rmANOVA shows significance for the NF in contrast to the CG, NF: *F*(2, 18) = 4.432, *P* = .019, Eta² = 0.198 (*M*_NF_(*t*0) = 7.70 (4.014), *M*_NF_(*t*1) = 6.2 (3.518), *M*_NF_(*t*2) = 5.05 (3.486), *N*_NF_ = 20), CG: *F*(2, 15) = 1.605, *P* = .236, Eta² = 0.186 (*M*_CG_(*t*0) = 5.82 (3.340), *M*_CG_(*t*1) = 5.71 (2.64), *M*_CG_(*t*2) = 5.24 (3.993), *N*_CG_ = 17). No group differences are found (*H* = 2.576, *P* = .278, see Table 2).

Regarding age effects, it is found that younger patients start the NF therapy with 2.2 points higher generalized anxiety than older patients and show a remission of 2.5 points (*M*_NF_ < 55*(t*1) = 7.3 (3.129), *M*_NF_ < 55*(t*2) = 4.8 (2.440), versus *M*_NF_ > 55 (*t*1) = 5.10 (3.695), *M*_NF_ > 55 (*t*2) = 5.30 (4.423)).

### Quality of Life

The rmANOVA for the NF intervention shows a significant increase of QoL due to the experimental intervention, in contrast to the CG, *F*(2, 18) = 3.593, *P* = .038, Eta² = 0.166 (*M*_NF_(*t*0) = 59.2 (21.742), *M*_NF_(*t*1) = 57.9 (23.633), *M*_NF_(*t*2) = 63.35 (22.843), *N*_NF_ = 20, Figure 4G).

The comparison of the delta values shows a trend (*H* = 5.279, *P* = .073, *d* = 0.606, see Table 2) indicating a higher increase of QoL for the NF compared to the CG.

There is no correlation for the treatment effect (∆*t*2 − *t*1) of QoL and the variables of psycho-oncological stress within the NF (see Supplemental Table 7).

### Self-efficacy

In contrast to the control intervention, the rmANOVA for changes in self-efficacy due to the NF intervention shows a significant main effect over time, *F*(2, 18) = 5.730, *P* = .007, Eta² = 0.241 (*M*_NF_(*t*0) = 26.9 (5.8017), *M*_NF_(*t*1) = 27.8 (6.035), Figure 4H). There is no effect for gender. There is a significant age effect, *F*(2, 15) = 12.846, *P* ≤ .001, Eta² = 0.631. No group differences are found (*H* = 0.296, *P* = .866, see Table 2).

Correlation analyses show a significant relation between self-efficacy and depression (rho = −.458, *P* = .048) for the NF intervention and a trend for the correlation with generalized anxiety (rho = −.436, *P* = .055). Correlations between self-efficacy and psycho-oncological symptom parameters are shown in Supplemental Table 8.

### Correlation Between Self-efficacy and Quality of Life

There is a high positive correlation of the NF treatment effect of QoL and self-efficacy measured with the GSE (rho = .564, *P* = .010).

The regression analysis of self-efficacy on QoL shows a positively prediction of self-efficacy on QoL, *F*(1) = 16.755, *P* ≤ .001, N = 20. The model provides an explained variance of 48.2% (see Supplemental Table 9).

## Discussion

Based on the results of a further review of the working group,^8^ the aim of this clinical RCT was to establish an NF therapy in psycho-oncology, to test its effectiveness. To our knowledge, this is the first investigation to compare NF with a mindfulness-based therapy, since it represents an already common used therapy option in order to deal with cancer-related symptoms.

Changes in frequency bands due to NF therapy were determined. Though, there were no changes in subjectively perceived cognitive impairment and fatigue in either the NF intervention or the mindfulness control group, all affective symptoms (distress, depression and anxiety) were shown to be significantly reduced due to both treatments. In addition, NF training increased self-efficacy and thus predicted QoL.

### Changes in Brain-wave Amplitudes During NF Sessions

Since the goal of the present study was to achieve relief from cancer-related symptoms such as cognitive impairment, stress, fatigue, depression, and anxiety, these goals were operationalized accordingly in the NF paradigm. Therefore, alpha-band and theta-beta ratios were trained in the current trial. The success of the training (eg, alpha) is assumed if symptom relief (arousal or distress) is evident. In the current RCT, a significant increase in alpha band from approximately 7 to above 8 Hz was achieved in 22 patients over the first 6 sessions in the first as well as in the second alpha band training (8-13 Hz). Thus, the effectiveness of the training can be assumed. Consequently, causal associations regarding this target are possible. Generally, an association between the increased alpha band and thus with increased relaxation ability and cancer-specific symptom relief can be hypothesized. To the best of our knowledge, no literature on psycho-oncological patients is available concerning the change of the theta and beta band. In the current study, no significant change in the theta-beta ratio corresponding to the target to 2.5 or <20 Hz was detected over the first 6 sessions. It is possible that a 5-minute training session was not effective enough across the 6 sessions. Unfortunately, there are no evidence-based data or training protocols in this cohort so far.

### No Changes in Perceived Cognitive Impairment in NF and Mindfulness Control

Even though Alvarez et al^7^ demonstrated a significant reduction in perceived cognitive impairment by a 10-week individualized NF training with 20 session (non-linear dynamical NF delivered using the Zengar NeurOptimal system), these results could not be replicated in the present study. The fact that no change in this parameter could be replicated may be due to the different NF system. However, there were no changes in subjectively perceived cognitive impairment in either the NF training or the mindfulness control group which might be due to the smaller number of therapy sessions. However, the present cohort showed significantly lower expressions in this parameter compared to a normative oncology cohort at all 3 measurement time points; thus it can be assumed that cognitive impairment does not seem to represent the expression and relevance to suffering in the present cohort.

### No Changes in Fatigue in NF and Mindfulness Control

The analyses of the present RCT showed no changes in mental fatigue measured with the MFI due to NF or mindfulness therapy intervention. However, a systematic review by Luctkar-Flude and Groll^6^ found a decrease in fatigue due to NF therapy. Another review article by Haller et al^41^ on cancer cohorts reported a decrease in fatigue due to mindfulness interventions. This suggests that in the present study mental fatigue measured with the MFI either did not validly represent the symptomatology of cancer patients, or in the present cohort, this was not subject to change—regardless of whether they received an NF or mindfulness intervention.

### Changes in Distress, Depression, and General Anxiety Due to NF and Mindfulness Therapy

Consistent with the review by Hetkamp et al^8^ a decrease in emotional distress and depression was hypothesized. Over the time, there was a significant decrease in these parameters for distress, depression, and generalized anxiety. It is clinically highly relevant that there was no difference between the less established NF therapy and an analogous clinically already very well accepted mindfulness intervention.^42,43^ According to the theoretical assumptions, the alleviation of affective symptoms may be associated with an observed alpha increase after NF training.^15^

What is apparent from the post-hoc analyses, however, is that the therapy intervention alone did not produce significant relief. Significance emerged only over time. It can be assumed that inclusion in the study and the in-depth medical history interview at baseline also had an impact on the alleviation of affective psycho-oncological symptoms. The fact that the significant effects were only achieved in combination with the anamnestic/first interview can also be interpreted as a therapeutic effect. The initial contact with the principal investigator can—and should in everyday clinical practice—serve as the first formation of a psychotherapeutic relationship.

### No Changes in Rumination in NF and Mindfulness Control

In the present study, no effects on rumination were found due to both NF therapy and mindfulness control. However, this contradicts previous studies, which report NF treatment effects on the expression of ruminative tendency in depressive persons,^44^ and mindfulness therapy effects on rumination.^23,24,45^ This is shown to be counterintuitive due to the high assumed association of depression and rumination.^46^ However, this survey also showed no correlation between the 2 variables, which may suggest that in this particular cohort, rumination propensity is not a suitable surrogate of affective mood state.

### Changes in Health-related Quality of Life (EORTC) Due to the NF Therapy

Consistent with the Iranian pilot study of breast cancer patients by Sarvghadi et al^9^ this current RCT demonstrated a significant increase in QoL over the time. Further analysis showed that this effect was stable only for the NF therapy. In accordance with Schmidt et al^10^ evidence for age effects was found. Interestingly, no significant increase in QoL was achieved through the mindfulness control. This makes it even more important to implement NF as a possible intervention offer to increase the QoL of cancer patients, which can be impacted by physiological and psychological cancer-related symptoms.^47-49^ Interestingly, even though previous studies suggest an association between psychological distress, depression, and QoL,^50-53^ no correlations between the increase in QoL with depression or the other variables of psycho-oncological distress were shown in the present study, which as such cannot be attributed to any theoretical basis so far. However, a strong correlation between cancer-specific QoL and self-efficacy was shown, as explicated below.

### Changes in Self-efficacy Due to the NF Intervention

In this RCT, the assumption according to Teufel et al^54^ could be confirmed that NF has an influence on self-efficacy. Self-efficacy refers to the belief in one’s own competences and the ability to achieve goals and succeed at difficult challenges. In cancer patients this might include reducing burdening symptoms by one’s own ability to achieve a relaxed state. Interestingly, patients in the current study did not experience any changes in self-efficacy due to mindfulness therapy intervention—contrary to the literature.^55^ However, there was a significant increase in self-efficacy as a result of the NF intervention. It is assumed that the acceptance and non-judgmental attitude fostered during mindfulness exercises can reduce negative emotions and increase self-efficacy in return.^5^ In many cases, self-efficacy is considered an important coping strategy after a cancer diagnosis or during cancer therapy.^56,57^ However, to date, this effect of NF has not been studied and/or uncovered in this cohort of patients. Accordingly, this RCT was the first to demonstrate an increase of self-efficacy with 5 weeks of NF therapy intervention in a cancer cohort. The results also suggest an association of self-efficacy with generalized anxiety and depression. The subject of further research should therefore be to investigate possible mediator/moderator effects in larger samples.

### Relationship Between QoL and Self-efficacy (GSE) in the NF Group

In accordance with previous literature,^56,58^ the present study found a relationship between QoL and self-efficacy, but only for the NF therapy intervention and not in case of the mindfulness control. In this study, self-efficacy was found being a predictor of QoL improvement due to the NF intervention. NF results in a significant increase in self-efficacy and thereby a significant increase in cancer-related QoL.

### Age Effects

Analyses of age effects showed no significance for the variable distress, but significance for those of depression and generalized anxiety. It could be demonstrated that younger patients benefited more in the NF intervention group, although they initially showed higher distress. The latter can be seen in line with the body of studies showing that young cancer patients face more severe obstacles than older ones.^59^ Some studies found additional benefits of NF in younger participants, such as improving executive function and maintained lateralized activity in stroke patients.^60,61^ A systematic review on predictors of NF efficacy showed no impact of age on training outcomes in most cases.^62^ However, none of these studies included cancer patients or depression and generalized anxiety as outcome variables.

### Limitations

During the first lockdown phase in Germany (March to May 2020), recruitment and data collection were completely paused, resulting in a high drop-out rate. Moreover, the chosen design as a waiting list parallel group study does not provide full elucidation of underlying mechanisms. Therefore, in the design of this study, an increase from this design to a cross-over design was discussed but discarded due to the high mortality of these patient groups. Furthermore, a higher degree of blinding would certainly have been beneficial. This advantage was accepted against the disadvantage of a possible investigator effect. Since many therapy studies suggest effects of therapists particularly with regard to their attachment styles and quality,^63^ the apparent limitation can be interpreted as a methodological and clinical strength.

### Clinical Implications

The analyses of this RCT not only demonstrate the effectiveness of NF at the symptom level, but also suggest a causal effect of alpha-band training as an index of relaxation ability and alleviation of affective symptoms in cancer patients. To date, this has been done only in single-case studies. This implies the need for further randomized controlled trials to generalize the results. Furthermore, studies are needed to investigate the effect of NF and mindfulness on parameters such as perceived cognitive impairment and fatigue in this cohort, as this is not the only study to show unambiguous findings. The effectiveness of NF on affective symptoms and QoL is unambiguous. The prevalence of affective symptoms and the general positive effect on the affective symptoms and QoL of psycho-oncological patients definitely underlines the use of NF as a further supportive measure in this cohort. This study suggests that NF alleviates affective symptoms in this patient group with similar results as the already established mindfulness practices. Therefore, this method should be made available to cancer patients according to patient inclinations and preferences. Considering that NF is significantly better at increasing self-efficacy in this cohort and that this increases QoL, further research on possible long-term effects is also of great interest.

## Conclusion

To the best of our knowledge, the current randomized controlled study is the first to investigate the technique of NF with regard to its effectiveness in a sample of cancer patients in comparison with an already established treatment in the field of psycho-oncology. Furthermore, for the first time, the changes in frequency bands due to training was determined in a large number of patients. There were no changes in subjectively perceived cognitive impairment and fatigue in either the NF therapy or the mindfulness control. Essential due to the high prevalence, all affective symptoms were shown to be significantly reduced due to the therapy, demonstrating the clinical relevance of this investigation. Moreover, it can be concluded that the NF therapy is not inferior to an analogous mindfulness intervention. While there is a need for further research on NF in this area, a clinically relevant benefit may be assumed due to the markedly high standardization and the high level of evidence due to the operationalization.

## Supplemental Material

sj-docx-1-ict-10.1177_15347354221149950 – Supplemental material for Neurofeedback Treatment Affects Affective Symptoms, But Not Perceived Cognitive Impairment in Cancer Patients: Results of an Explorative Randomized Controlled Trial Click here for additional data file. Supplemental material, sj-docx-1-ict-10.1177_15347354221149950 for Neurofeedback Treatment Affects Affective Symptoms, But Not Perceived Cognitive Impairment in Cancer Patients: Results of an Explorative Randomized Controlled Trial by Madeleine Fink, Saskia Pasche, Kira Schmidt, Mitra Tewes, Martin Schuler, Bernhard W. Mülley, Dirk Schadendorf, Norbert Scherbaum, Axel Kowalski, Eva-Maria Skoda and Martin Teufel in Integrative Cancer Therapies
