# Supplementary material for: Neurofeedback Treatment Affects Affective Symptoms, But Not Perceived Cognitive Impairment in Cancer Patients: Results of an Explorative Randomized Controlled Trial
Source: Integr Cancer Ther. 2023 Jan 24;22:15347354221149950. doi: 10.1177/15347354221149950 (PMC9893099; doi:10.1177/15347354221149950)
Supplement: sj-docx-1-ict-10.1177_15347354221149950 – Supplemental material for Neurofeedback Treatment Affects Affective Symptoms, But Not Perceived Cognitive Impairment in Cancer Patients: Results of an Explorative Randomized Controlled Trial [file sj-docx-1-ict-10.1177_15347354221149950.docx]

# Supplements

Table S1. *Descriptive statistics*

|  | *Experimental Group*  *Neurofeedback* | | | | | | |  | *Control Group*  *Mindfulness* | | | | | | |  |  |
| --- | --- | --- | --- | --- | --- | --- | --- | --- | --- | --- | --- | --- | --- | --- | --- | --- | --- |
| Outcome | *N** | *M* | *SD* | *S (SE)* | *K (SE)* | *min* | *max* |  | *N** | *M* | *SD* | *S (SE)* | *K (SE)* | *min* | *max* |  |  |
| PCI t0 | 21 | 47.9 | 15.313 | -.240 (.501) | -1.298 (.972) | 23 | 69 |  | 21 | 48.43 | 14.116 | -.991 (.501) | .623 (.972) | 16 | 68 |  |  |
| PCI t1 | 21 | 51.33 | 15.793 | -1.008 (.501) | .595 (.972) | 15 | 69 |  | 20 | 50.15 | 12.946 | -.979 (5.12) | .805 (.992) | 20 | 68 |  |  |
| PCI t2 | 20 | 53.4 | 14.478 | -.378 (.512) | -1.004 (.992) | 25 | 72 |  | 20 | 49.25 | 16.251 | -.915 (.512) | -253 (.992) | 17 | 67 |  |  |
| PCI Δt1-t0 | 21 | 3.429 | 11.241 | 1.934 (.501) | 5.482 (.972) | -9 | 41 | Z = -.906;  p = .365 | 20 | 1.8 | 7.77 | 1.4489 (.512) | 3.111 (.992) | -7 | 25 | Z = -.705;  p = .481 |  |
| PCI Δt2-t1 | 20 | .35 | 8.993 | -.455 (.512) | .069 (.992) | -19 | 15 |  | 19 | -1.158 | 13.2 | -1.649 (.524) | 5.33 (1.014) | -43 | 21 |  | U = 185;  p = .888 |
| MFI t0 | 21 | 12 | 1.183 | 0 (.501) | -.89 (.970) | 10 | 14 |  | 20 | 11.45 | .999 | -1.433 (.512) | 2.204 (.992) | 9 | 13 |  |  |
| MFI t1 | 21 | 11.81 | 1.167 | .407 (.501) | -.633 (9.72) | 10 | 14 |  | 18 | 11.56 | .984 | -.173 (.536) | -.802 (1.038) | 10 | 13 |  |  |
| MFI t2 | 19 | 12.11 | 1.524 | .119 (.524) | -.804 (1.014) | 10 | 15 |  | 21 | 11.48 | 1.167 | -.041 (.501) | .282 (.972) | 9 | 14 |  |  |
| MFI Δt1-t0 | 21 | -.191 | 1.209 | -.724 (.501) | -.286 (.972) | -3 | 1 | Z = -1.159;  p = .246 | 20 | -.1 | 2.174 | -.712 (.512) | .104 (.992) | -5 | 3 | Z = -.075;  p = .940 |  |
| MFI Δt2-t1 | 20 | .55 | 2.35 | -.06 (.512) | -.503 (.992) | -4 | 5 |  | 20 | -.05 | 2.212 | .135 (.512) | .954 (.992) | -5 | 5 |  | U = 170;  p = .409 |
| DT t0 | 21 | 6.19 | 2.04 | -.48 (.501) | -.834 (.972) | 2 | 9 |  | 20 | 6.3 | 2.342 | -.566 (.512) | -.641 (.992) | 2 | 10 |  |  |
| DT t1 | 18 | 6 | 2.401 | -.287 (.536) | -.399 (1.038) | 1 | 10 |  | 19 | 6.37 | 2.033 | -.43 (.524) | -.006 (1.014) | 2 | 10 |  |  |
| DT t2 | 19 | 5.89 | 2.644 | -.337 (.524) | -1.146 (1.014) | 2 | 10 |  | 19 | 5.53 | 2.27 | -.580 (.524) | -.578 (1.014) | 1 | 9 |  |  |
| DT Δt1-t0 | 18 | -.278 | 1.994 | -.119 (.536) | -1.017 (1.038) | -4 | 3 | Z = -.087;  p = .930 | 18 | -.167 | 1.653 | -.141 (.536) | -.422 (1.038) | -3 | 3 | Z = -.486;  p = .627 |  |
| DT Δt2-t1 | 18 | -.0556 | 2.578 | 1.738 (.536) | 5.22 (1.038) | -4 | 8 |  | 18 | -.667 | 2.169 | -.367 (.536) | -.539 (1.038) | -5 | 3 |  | U = 153.5; p = .785 |
| PHQ-8 t0 | 20 | 8.2 | 4.549 | .825 (.512) | .154 (.992) | 2 | 19 |  | 21 | 9.1 | 5.049 | 1.25 (.501) | 1.1736 (.972) | 3 | 23 |  |  |
| PHQ-8 t1 | 20 | 7.55 | 4.286 | .554 (.512) | -.407 (.992) | 1 | 17 |  | 18 | 7.56 | 3.468 | .784 (.536) | .536 (1.038) | 3 | 16 |  |  |
| PHQ-8 t2 | 20 | 6.6 | 4.235 | .180 (.512) | -1.07 (.992) | 1 | 15 |  | 21 | 7.19 | 5.076 | 1.173 (.501) | 1.079 (.972) | 1 | 20 |  |  |
| PHQ-8 Δt1-t0 | 20 | -.85 | 2.72 | -.507 (.512) | 1.275 (.992) | -8 | 4 | Z = -.524;  p = .6 | 20 | -.6 | 3.691 | .198 (.512) | 1.682 (.992) | -9 | 8 | Z = -.438;  p = .661 |  |
| PHQ-8 Δt2-t1 | 19 | -.5263 | 2.568 | .99 (.524) | 3.494 (1.014) | -5 | 7 |  | 20 | -1.2 | 4.098 | -2.032 (.512) | 6.347 (.992) | -15 | 5 |  | U = 187.5;  p = .943 |
| RSQ t0 | 21 | 41.05 | 9.724 | .263 (.501) | .125 (.972) | 23 | 63 |  | 21 | 40.05 | 10.703 | .755 (.501) | -.118 (.972) | 27 | 64 |  |  |
| RSQ t1 | 21 | 39.38 | 10.961 | .456 (.501) | -.686 (.972) | 24 | 63 |  | 20 | 38.5 | 12.395 | .759 (.512) | -.528 (.992) | 21 | 62 |  |  |
| RSQ t2 | 19 | 36.37 | 9.604 | .496 (.524) | -.997 (1.014) | 22 | 53 |  | 21 | 37.48 | 10.4 | .722 (.501) | -.07 (.972) | 21 | 58 |  |  |
| RSQ Δt1-t0 | 21 | -1.667 | 6.248 | .095 (.501) | -.359 (.972) | -13 | 11 | Z = -.806;  p = .42 | 20 | -1.35 | 4.475 | -.143 (.512) | -1.411 (.992) | -9 | 5 | Z = -.436;  p = .663 |  |
| RSQ Δt2-t1 | 19 | -2.368 | 9.539 | -2.781 (.524) | 10.274 (1.014) | -37 | 9 |  | 20 | -.7 | 7.463 | -1.128 (.512) | 3.262 (.992) | -23 | 11 |  | U = 178;  p = .735 |
| GAD-7 t0 | 21 | 7.76 | 3.923 | 1.109 (.501) | .540 (.972) | 3 | 17 |  | 21 | 7.33 | 4.963 | 1.058 (.501) | .048 (.972) | 2 | 19 |  |  |
| GAD-7 t1 | 21 | 6.62 | 3.930 | .812 (.501) | .281 (.972) | 1 | 15 |  | 17 | 5.71 | 4.79 | 1.272 (.524) | 1.249 (1.014) | 2 | 12 |  |  |
| GAD-7 t2 | 20 | 5.05 | 3.486 | .376 (.512) | -.612 (.992) | 0 | 12 |  | 19 | 4.79 | 4.008 | 1.272 (.524) | 1.249 (1.014) | 0 | 14 |  |  |
| GAD-7 Δt1-t0 | 21 | -1.143 | 3.071 | -.149 (.501) | 1.379 (.972) | -8 | 6 | Z = -.343;  p =.732 | 20 | .1 | 2.292 | -1.418 (.512) | 2.354 (992) | -6 | 3 | Z = -1.927;  p = .054 |  |
| GAD-7 Δt2-t1 | 20 | -1.15 | 3.048 | .121 (.512) | .524 (.992) | -8 | 5 |  | 20 | -1.15 | 4.308 | -1.115 (.512) | 3.771 (.992) | -14 | 7 |  | U = 176;  p = .512 |
| EORTC t0 | 21 | 58.76 | 21.286 | -.804 (.501) | -.589 (.972) | 17 | 83 |  | 21 | 50.81 | 19.676 | .144 (.501) | -.034 (.972) | 17 | 92 |  |  |
| EORTC t1 | 21 | 57.52 | 23.099 | -.477 (.501) | -.455 (.972) | 8 | 92 |  | 20 | 52.05 | 24.451 | -.764 (.512) | -.283 (.992) | 0 | 83 |  |  |
| EORTC t2 | 20 | 63.35 | 22.843 | -.167 (.512) | -1.07 (.992) | 25 | 100 |  | 21 | 57.59 | 22.024 | -.509 (.501) | -.281 (.997) | 8 | 92 |  |  |
| EORTC Δt1-t0 | 21 | -1.238 | 19.679 | -.755 (.501) | 3.542 (.972) | -59 | 42 | Z = -1.597;  p = .11 | 20 | 1.2 | 17.404 | 1.141 (.512) | 1.114 (.992) | -25 | 41 | Z = -.463;  p = .643 |  |
| EORTC Δt2-t1 | 20 | 5.45 | 21.717 | -1.351 (.512) | 3.361 (.992) | -58 | 42 |  | 20 | 4.667 | 21.211 | 1.118 (.512) | 3.13 (.992) | -34 | 67 |  | U = 169;  p = .396 |
| GSE t0 | 21 | 26.57 | 5.921 | -.994 (.501) | .692 (.972) | 12 | 36 |  | 21 | 28.76 | 4.898 | -.772 (.501) | 1.494 (.972) | 16 | 38 |  |  |
| GSE t1 | 21 | 27.52 | 6.013 | -.932 (.501) | .806 (.992) | 14 | 38 |  | 20 | 28.3 | 4.635 | -.403 (.512) | -.346 (.992) | 19 | 36 |  |  |
| GSE t2 | 20 | 28.05 | 5.434 | -1.277 (.512) | 2.219 (.992) | 13 | 37 |  | 20 | 29.14 | 6.247 | -.815 (.512) | .041 (.992) | 13 | 38 |  |  |
| GSE Δt1-t0 | 21 | .9534 | 2.872 | 1.299 (.501) | 2.18 (.972) | -4 | 8 | Z = -.156;  p = .876 | 20 | -.4 | 3.545 | -.578 (.512) | .041 (.992) | -8 | 5 | Z = -.153;  p = .879 |  |
| GSE Δt2-t1 | 20 | .25 | 3.416 | -1.038 (.512) | 3.484 (.992) | -10 | 6 |  | 20 | .4 | 4.382 | .067 (.512) | -.128 (.992) | -7 | 9 |  | U = 181;  p = .620 |

*Notes.* *N* = 42 (*n* = 21; *n* = 21). * Statistical outliers ± one SD. PCI = Perceived Cognitive Impairments measured by FACT-Cog. MFI = Mental Fatigue measured by MFI-20; DT = Distress Thermometer; PHQ-8 = Depression measured by Patient Health Questionnaire Depression Scale; RSQ = Rumination measured by Ruminative Response Scale of Response Styles Questionnaire; GAD-7 = Generalized Anxiety Disorder Scale-7; EORTC = Cancer-related Life Quality measured by European Organization for Research and Treatment of Cancer Core Quality of Life Questionnaire (EORTC QLQ-C30); GSE = General Self-Efficacy Scale. All analyses were conducted with the Wilcoxon-Test (Z) and Mann-Whitney-U-Test.

Table S2. *Mean values of the target parameters 60 seconds of the respective training*

|  | *First Alpha-Training* | | *Second Alpha-Training* | | *Theta/Beta-Training* | |
| --- | --- | --- | --- | --- | --- | --- |
| Session | *N* | *M (SD)* | *N* | *M (SD)* | *N* | *M (SD)* |
| 1 | 15 | 7.233 (2.038) | 15 | 6.736 (2.869) | 14 | 20.282 (4.42) |
| 2 | 15 | 7.56 (2.417) | 15 | 7.055 (2.411) | 14 | 19.111 (4.703) |
| 3 | 15 | 7.321 (2.043) | 15 | 7.803 (2.595) | 14 | 19.939 (5.232) |
| 4 | 15 | 7.618 (2.254) | 15 | 7.308 (3.399) | 14 | 19.992 (6.761) |
| 5 | 15 | 8.9 (4.107) | 15 | 8.675 (3.157) | 14 | 19.342 (4.514) |
| 6 | 15 | 8.833 (3.612) | 15 | 8.934 (2.7) | 14 | 20.357 (4.711) |

*Notes.* Target parameter for alpha training was defined as 8 - 13 Hz; for theta/beta training, < 20 Hz (ratio of 2.5).

Table S3. *Comparing the FACT-Cog scores of this cohort with a normative psycho-oncological sample^29^*

|  | FACT-Cog | | | | |
| --- | --- | --- | --- | --- | --- |
|  | M (SD) | N | T | p | Confidence Intervall |
| t0 | 48.17 (14.548) | 42 | -3.846 | ≤.001 | -13.17; -4.10 |
| t1 | 50.76 (14.307) | 41 | -2.705 | ≤.01 | -10.56; -1.53 |
| t2 | 51.33 (15.336) | 40 | -2.258 | ≤.05 | -10.38; -.57 |

*Note.* Test value = 56.8 (11.2).

**Table S4.** *Prevalence of distress before (t0) and after (t1) waitlist, and after intervention (t2).*

|  | DT | | |
| --- | --- | --- | --- |
|  | t0 | t1 | t2 |
| < 5 | 12 (29,3%) | 8 (21,3%) | 10 (26,3%) |
| ≥ 5 | 29 (70,7%) | 29 (78,7%) | 28 (73,7%) |
| *N* | 41 | 37 | 38 |

*Notes.* DT = Distress Thermometer, scores ≥ 5 indicate high distress.

**Table S5.** *Prevalence of depression (PHQ-8) before (t0) and after (t1) waitlist and after intervention (t2)*.

|  | PHQ-8 | | |
| --- | --- | --- | --- |
|  | t0 | t1 | t2 |
| < 10 | 27 (65.9%) | 28 (73.7%) | 29 (70.7%) |
| ≥ 10 | 14 (34.1%) | 10 (26.3%) | 12 (29.3%) |
| *N* | 41 | 38 | 41 |

*Notes.* PHQ-8 = Patient Health Questionnaire-8, scores ≥ 10 indicate Major Depression.

**Table S6.** *Prevalence of generalized anxiety (GAD-7) before (t0) and after (t1) waitlist and after intervention (t2).*

|  | GAD-7 | | |
| --- | --- | --- | --- |
|  | t0 | t1 | t2 |
| < 5 | 31 (73,8%) | 34 (89,5%) | 35 (89,7%) |
| ≥ 5 | 11 (26,2%) | 4 (10,5%) | 4 (10,3%) |
| *N* | 42 | 38 | 39 |

*Notes.* GAD-7 = Generalized anxiety, scores ≥ 5 indicated severely high anxiety.

**Table S7*.*** *Spearman’s correlation between quality of life and psycho-oncological symptom parameters for the treatment effect ∆t2–t1 within the experimental group.*

|  | FACT-Cog | MFI | PHQ | GAD | DT | RSQ |
| --- | --- | --- | --- | --- | --- | --- |
| *rho*  Quality of life  (EORTC QLQ-C30) | -.357 | .371 | -.085 | -.100 | .039 | -.172 |
| *p (two-sided)* | .123 | .107 | .731 | .675 | .878 | .482 |
| *N* | 20 | 20 | 19 | 20 | 18 | 19 |

*Note.* Two-sided.

**Table S8.** *Spearman’s correlation between self-efficacy and psycho-oncological symptom parameters for the treatment effect ∆t2–t1 within the experimental group.*

|  | FACT-Cog | MFI | PHQ | GAD | DT | RSQ |
| --- | --- | --- | --- | --- | --- | --- |
| *rho*  Self-efficacy | -.085 | .129 | -.458 | -.436 | -.147 | -.155 |
| *p (two-sided)* | .720 | .587 | .048 | .055 | .560 | .527 |
| *N* | 20 | 20 | 19 | 20 | 18 | 19 |

*Note.* Two-sided.

**Table S9.** *Regression analysis of self-efficacy on quality of life.*

| Predictor | *β* | *βse* | *t* | *p* |
| --- | --- | --- | --- | --- |
| *Intercept* | 4,347 |  | 1,207 | ,234 |
| Self-efficacy | 4,414 | ,694 | 4,093 | ,001 |

*Notes.* Total *R²* = .482, *F*(1) = 16.755, *p* ≤ .001, *N* = 20.

**Case number calculation.** The effort of an individualized intervention in the patients' daily clinical routine and the temporal inclusion in a group therapy were considered in the calculation. Based on a recruitable sample (before the COVID-19 pandemic) of N = 2 x 40 subjects, we calculate the width of the 95% confidence interval for the group difference in FACT-Cog scores.^1^ Calculations were performed in PASS 13 ("Confidence Intervals for the Difference between Two Means").^2^ A clinically relevant difference of approximately eleven points is suggested in Bell et al.^3^.

1. Bonomi A, Cella D, Hahn E, et al. Multilingual translation of the Functional Assessment of Cancer Therapy (FACT) quality of life measurement system. *Quality of Life research*. 1996;5(3):309-320.

2. Hintze J. PASS 13. NCSS, LLC, Kaysville, UT, USA. 2014.

3. Bell M, Dhillon H, Bray V, Vardy J. Important differences and meaningful changes for the functional assessment of cancer therapy-cognitive function (FACT-Cog). *Journal of Patient-Reported Outcomes*. 2018;2(1):1-11.

**
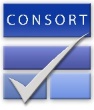
CONSORT 2010 checklist of information to include when reporting a randomised trial***

| **Section/Topic** | **Item No** | **Checklist item** | **Reported on page No** |
| --- | --- | --- | --- |
| **Title and abstract** | | | |
|  | 1a | Identification as a randomised trial in the title | 1 |
|  | 1b | Structured summary of trial design, methods, results, and conclusions (for specific guidance see CONSORT for abstracts) | 2 |
| **Introduction** | | | |
| Background and objectives | 2a | Scientific background and explanation of rationale | 4-7 |
|  | 2b | Specific objectives or hypotheses | 7 |
| **Methods** | | | |
| Trial design | 3a | Description of trial design (such as parallel, factorial) including allocation ratio | 7-8 |
|  | 3b | Important changes to methods after trial commencement (such as eligibility criteria), with reasons | - |
| Participants | 4a | Eligibility criteria for participants | 7-8 |
|  | 4b | Settings and locations where the data were collected | 7 |
| Interventions | 5 | The interventions for each group with sufficient details to allow replication, including how and when they were actually administered | 8-10 |
| Outcomes | 6a | Completely defined pre-specified primary and secondary outcome measures, including how and when they were assessed | 11-13 |
|  | 6b | Any changes to trial outcomes after the trial commenced, with reasons | - |
| Sample size | 7a | How sample size was determined | See above |
|  | 7b | When applicable, explanation of any interim analyses and stopping guidelines |  |
| Randomisation: |  |  |  |
| Sequence generation | 8a | Method used to generate the random allocation sequence | 8 |
|  | 8b | Type of randomisation; details of any restriction (such as blocking and block size) | 8 |
| Allocation concealment mechanism | 9 | Mechanism used to implement the random allocation sequence (such as sequentially numbered containers), describing any steps taken to conceal the sequence until interventions were assigned | 8 |
| Implementation | 10 | Who generated the random allocation sequence, who enrolled participants, and who assigned participants to interventions | 7-8 |
| Blinding | 11a | If done, who was blinded after assignment to interventions (for example, participants, care providers, those assessing outcomes) and how | 8 |
|  | 11b | If relevant, description of the similarity of interventions | 10-11 |
| Statistical methods | 12a | Statistical methods used to compare groups for primary and secondary outcomes | 13-15 |
|  | 12b | Methods for additional analyses, such as subgroup analyses and adjusted analyses | 13-15 |
| **Results** | | | |
| Participant flow (a diagram is strongly recommended) | 13a | For each group, the numbers of participants who were randomly assigned, received intended treatment, and were analysed for the primary outcome | 15 |
|  | 13b | For each group, losses and exclusions after randomisation, together with reasons | 15 |
| Recruitment | 14a | Dates defining the periods of recruitment and follow-up | 15 |
|  | 14b | Why the trial ended or was stopped | 7 |
| Baseline data | 15 | A table showing baseline demographic and clinical characteristics for each group | 15 |
| Numbers analysed | 16 | For each group, number of participants (denominator) included in each analysis and whether the analysis was by original assigned groups | 15 |
| Outcomes and estimation | 17a | For each primary and secondary outcome, results for each group, and the estimated effect size and its precision (such as 95% confidence interval) | 15-23 |
|  | 17b | For binary outcomes, presentation of both absolute and relative effect sizes is recommended | 15-23 |
| Ancillary analyses | 18 | Results of any other analyses performed, including subgroup analyses and adjusted analyses, distinguishing pre-specified from exploratory | 15-23 and above |
| Harms | 19 | All important harms or unintended effects in each group (for specific guidance see CONSORT for harms) |  |
| **Discussion** | | | |
| Limitations | 20 | Trial limitations, addressing sources of potential bias, imprecision, and, if relevant, multiplicity of analyses | 28-29 |
| Generalisability | 21 | Generalisability (external validity, applicability) of the trial findings | 29-30 |
| Interpretation | 22 | Interpretation consistent with results, balancing benefits and harms, and considering other relevant evidence | 23-31 |
| **Other information** | | |  |
| Registration | 23 | Registration number and name of trial registry | 3 |
| Protocol | 24 | Where the full trial protocol can be accessed, if available | 7 |
| Funding | 25 | Sources of funding and other support (such as supply of drugs), role of funders | 3 and 31 |

*We strongly recommend reading this statement in conjunction with the CONSORT 2010 Explanation and Elaboration for important clarifications on all the items. If relevant, we also recommend reading CONSORT extensions for cluster randomised trials, non-inferiority and equivalence trials, non-pharmacological treatments, herbal interventions, and pragmatic trials. Additional extensions are forthcoming: for those and for up to date references relevant to this checklist, see [www.consort-statement.org](http://www.consort-statement.org)
